# Foreign body granuloma masquerading as malignant blue nevus

**DOI:** 10.1016/j.jdcr.2025.04.040

**Published:** 2025-05-30

**Authors:** Richard Rookwood, Nicole Schiraldi, Tian Ran Zhu, Adam Chahine, David Ciocon

**Affiliations:** a Division of Dermatology, Department of Medicine, Montefiore Medical Center, Albert Einstein College of Medicine, Bronx, New York; b Clinic 5C, Spokane, Washington

**Keywords:** blue nevus-like melanoma, clinical mimicker, foreign body granuloma, foreign body reaction, malignant blue nevus, pigmented lesion, traumatic tattoo

## Case description

**Fig 1:**
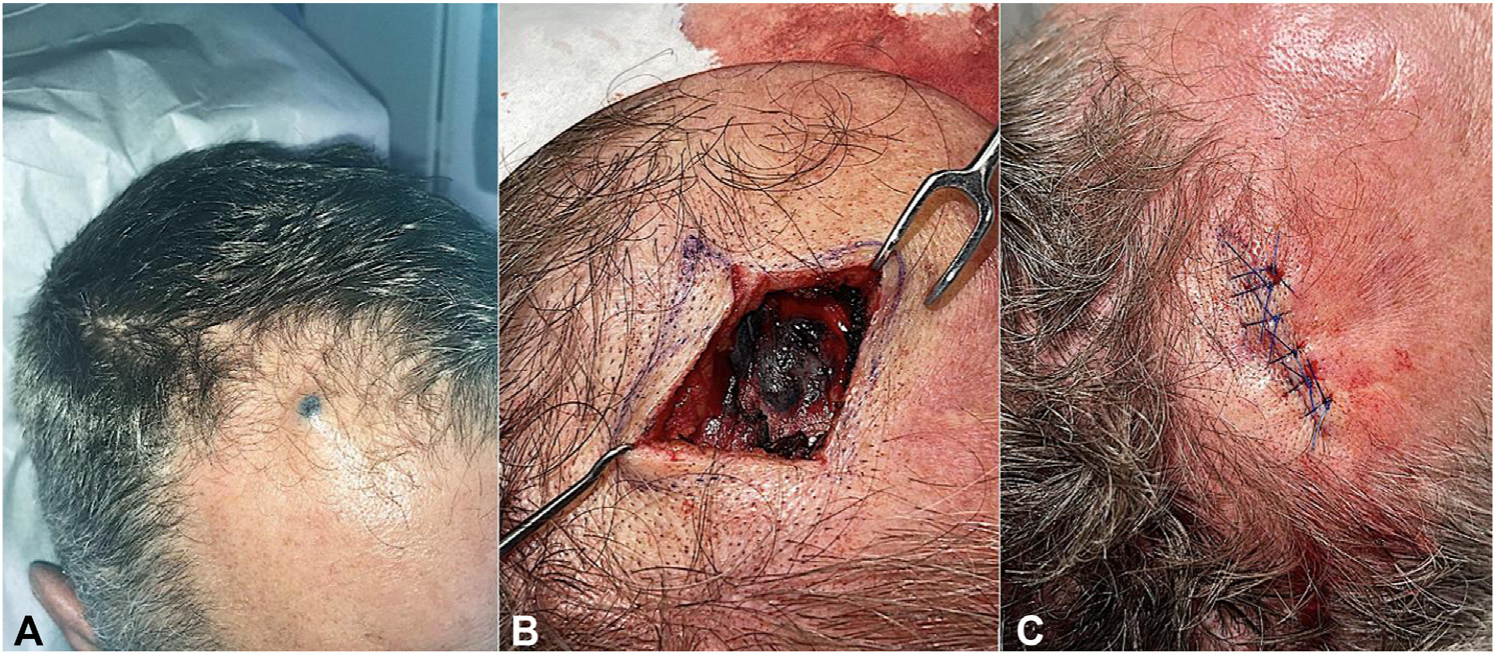
On the right frontal scalp is a 1 cm firm fixed blue-black papulonodule (**A**). Exposure of the frontal scalp reveals blue-black pigment invading beyond subcutaneous fat to galea aponeurotica (**B**). Primary linear closure of frontal scalp defect after surgical exploration and biopsy (**C**).

A 51-year-old healthy male presented with an asymptomatic blue papule on the right frontal scalp. The blue lesion had been present since early childhood and remained stable until a few months ago, when it began to gradually enlarge. Examination revealed a 1 cm solitary, firm, round, blue papulonodule (Fig 1, *A*). The patient denied recent trauma, bleeding, oozing, or ulceration at the site of the lesion and reported no personal or family history of melanoma or other cutaneous keratinocyte carcinomas.

**Question 1: What is the diagnosis?**

A. Malignant blue nevus
B. Pigmented basal cell carcinoma
C. Nodular melanoma
D. Foreign body granuloma
E. Merkel cell carcinoma

**Answers:**

**Fig 2:**
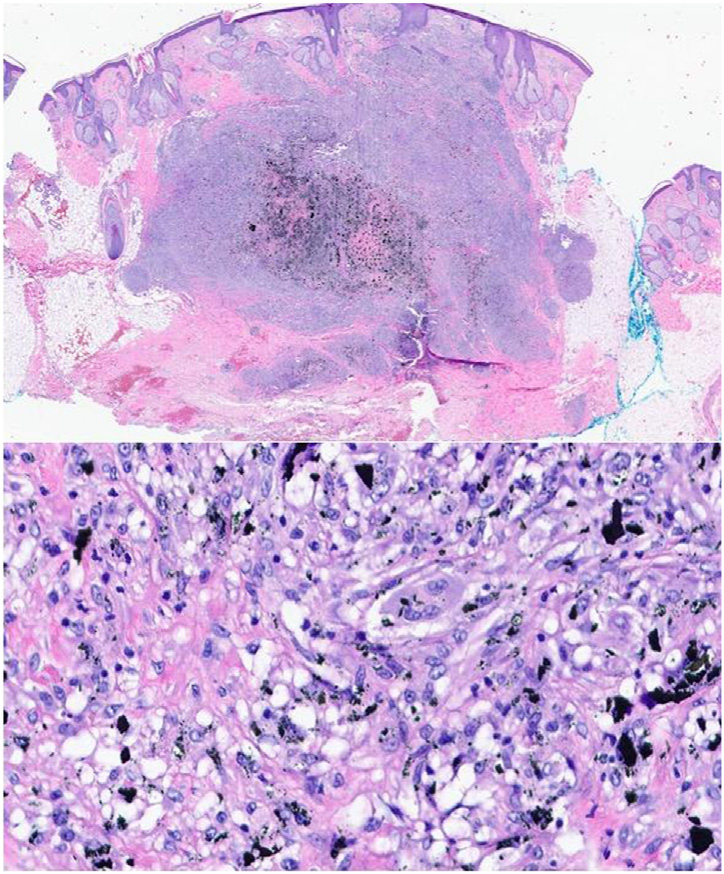
Dermal black pigment deposits with surrounding granulomatous reaction and multinucleated giant cells. Hematoxylin and eosin stain, magnification 20× (*top*) and 100× (*bottom*).
E. Merkel cell carcinoma—Incorrect.

A. Malignant blue nevus—Incorrect.
B. Pigmented basal cell carcinoma—Incorrect.
C. Nodular melanoma—Incorrect.
D. Foreign body granuloma—Correct. Clinicopathologic correlation supports diagnosis of foreign body granuloma. The initial presentation of a firm, enlarging, blue-black papulonodule on the scalp raises suspicion for malignancy. Intraoperatively, the pigmented lesion extended to the galea aponeurotica (Fig 1, *B*). Given the degree of invasion and potential subperiosteal involvement, the procedure was halted in favor of obtaining tissue for histopathologic assessment (Fig 1, *C*). Hematoxylin and eosin-stained tissue demonstrated dermal deposits of foreign body pigment associated with lymphohistiocytic infiltrate and multi-nucleated giant cells without evidence of malignancy (Fig 2). Upon further inquiry, the patient revealed a childhood history of trauma, whereby ink from a fountain pen was inoculated at the injury site on the right frontal scalp.

## Discussion

Traumatic tattoos are foreign body reactions resulting from pigment deposition in the dermis that becomes trapped if not removed before re-epithelialization.^1^ These reactions are often secondary to penetrating injuries, as evidenced by our patient, who developed a pigmented lesion following a breached skin barrier and deposition of ink from a fountain pen. Traumatic tattoos, regardless of etiology, often present as blue or black-pigmented lesions due to a phenomenon known as the Tyndall effect, where blue light is preferentially reflected by dermally embedded pigmented particles, as can also be seen in blue nevus.^2^ In this case, the Tyndall effect was caused by the physical deposition of the ink itself. Though usually stable, changes in these lesions can occur after many years, likely due to immune reactivation and renewed recognition of retained foreign material.

Enlarging foreign body granulomas can mimic malignant blue nevus (MBN), a rare and aggressive variant of melanoma. MBN most often arises on the scalp and presents as a progressively enlarging blue-black papule, nodule, or plaque. Dermoscopy may aid in distinguishing these entities: traumatic tattoos appear as homogeneous blue-gray lesions without structure or vascularity, while MBN typically shows asymmetry, multicolor pigmentation, irregular blotches, regression structures, and atypical vessels.^3^ Histopathologically, foreign body granulomas contain nonmelanin pigment and granulomatous inflammation in the absence of spindled or epitheliod melanocytes. In contrast, MBN shows dermal proliferation of pigmented spindled melanocytes with varying degrees of mitosis, atypia, and deep invasion with positive immunostains (S100, SOX10, HMB45, MelanA, MART1).^4^

This case illustrates how foreign body granulomas can clinically resemble malignancy, and underscores the importance of careful history taking, clinicopathologic correlation, and operative prudence in the evaluation and management of suspected malignant blue

## Conflicts of interest

None disclosed.

## References

[1] Park J.S., Min J.H. (2016). Effective prevention of posttraumatic tattoo using hydrosurgical debridement in the ED. Am J Emerg Med.

[2] Navarrete-Dechent C., Abarzúa-Araya A., Andreani S., Villaseca M.A. (2023). Traumatic tattoo as a clinical and dermoscopic mimicker of pigmented basal cell carcinoma and other tumors: a series of three cases. Int J Dermatol.

[3] Lallas K., Arceu M., Martinez G. (2022). Dermoscopic predictors of benignity and malignancy in equivocal lesions predominated by blue color. Dermatology.

[4] Choe S., Moroz M., Puvvadi S. (2024). Malignant blue nevus: epidemiology, socioeconomic factors, and disease presentation. J Clin Oncol.

